# Comparative Metabolomic Profiling of Amino Acids in Dried Blood Spots from Dichorionic and Monochorionic Twins Complicated by Selective Fetal Growth Restriction and Twin-to-Twin Transfusion Syndrome

**DOI:** 10.3390/ijms27219428

**Published:** 2026-10-22

**Authors:** Vitaliy V. Chagovets, Natalia A. Frankevich, Alisa O. Tokareva, Anastasia V. Novoselova, Marina N. Yushina, Fadi Hajjar, Tamara E. Karapetyan, Irina I. Ryumina, Vladimir E. Frankevich, Gennady T. Sukhikh

**Affiliations:** 1 National Medical Research Center for Obstetrics Gynecology and Perinatology Named After Academician V.I. Kulakov of the Ministry of Healthcare of Russian Federation, 117997 Moscow, Russia; v_chagovets@oparina4.ru (V.V.C.); a_tokareva@oparina4.ru (A.O.T.); a_novoselova@oparina4.ru (A.V.N.); m_yushina@oparina4.ru (M.N.Y.); f_hajjar@oparina4.ru (F.H.); t_karapetyan@oparina4.ru (T.E.K.); i_ryumina@oparina4.ru (I.I.R.); v_frankevich@oparina4.ru (V.E.F.); g_sukhikh@oparina4.ru (G.T.S.); 2 Department of Obstetrics, Gynecology, Perinatology and Reproductology, Institute of Professional Education, Federal State Autonomous Educational Institution of Higher Education I.M. Sechenov First Moscow State Medical University of the Ministry of Health of the Russian Federation, 119991 Moscow, Russia

**Keywords:** dried blood spots, amino acids, twins, twin-to-twin transfusion syndrome, fetal growth restriction, metabolomics, high-performance liquid chromatography-tandem mass spectrometry

## Abstract

Multiple pregnancy is associated with a high risk of perinatal complications, among which twin-to-twin transfusion syndrome (TTTS) and selective fetal growth restriction (sFGR) are of particular importance. These conditions are specific to monochorionic twins, yet their metabolic consequences in newborns remain poorly understood. Dried blood spots (DBSs) represent a convenient and non-invasive biomaterial for metabolomic analysis. This study included 7 twin pairs with sFGR, 10 pairs with TTTS, and 16 pairs from uncomplicated pregnancies. The amino acid profile in DBSs was determined by HPLC–MS/MS. Statistical significance of variation in normalized intra-pair difference in amino acid levels (ΔS_twins) was assessed using the Mann–Whitney U test with Benjamini–Hochberg false discovery rate correction. The dynamics of ΔS_twins between birth and the corrected full-term age were analysed. In comparing sFGR group with controls, intra-pair differences for glycine and alanine were altered at birth, and for β-alanine and leucine at the second time point. In the TTTS group, deviations from controls were observed for glutamine (at the second time point) and for alanine, α-aminobutyric acid, and aspartic acid (in terms of dynamics). Direct comparison between the two pathologies revealed differences in citrulline (TTTS vs. sFGR at birth) and in several amino acids over time (valine, leucine, ornithine). Dynamic analysis further showed that placental type significantly influenced the postnatal metabolic reorganisation of valine and alanine. These preliminary observations require independent validation and should not be considered validated clinical biomarkers.

## 1. Introduction

Twin pregnancy is associated with an increased risk of maternal and perinatal complications compared with singleton gestations [1]. Among the most significant are preterm delivery, preeclampsia (PE), gestational diabetes mellitus (GDM), as well as conditions specific to monochorionic (MC) twins, namely twin-to-twin transfusion syndrome (TTTS) and selective fetal growth restriction (sFGR) of one fetus [2]. The incidence of TTTS among MC twin pregnancies reaches 10–15%, while sFGR occurs even more frequently, substantially affecting neonatal morbidity and mortality [3]. Traditional antenatal monitoring tools, including ultrasound fetometry and Doppler assessment of fetal and placental vessels, allow these conditions to be diagnosed only at advanced stages, when pathological changes have already become irreversible. Consequently, there is growing interest in the search for early biomarkers capable of reflecting metabolic disturbances at a preclinical level.

In this context, metabolomics—the comprehensive study of low-molecular-weight metabolites (amino acids, lipids, organic acids, etc.)—represents a powerful approach to understanding the pathogenesis of multiple-pregnancy complications [4]. The metabolome is the downstream endpoint of the genome-transcriptome-proteome cascade and most accurately mirrors the current physiological state of the organism, responding to pathological processes more rapidly than clinical symptoms [5]. Metabolomic studies in TTTS, performed on amniotic fluid, maternal blood, and cord blood, have revealed substantial alterations in amino acid, lipid, and organic acid profiles. The donor fetus often exhibits a metabolic pattern consistent with insufficient nutrient supply and oxidative stress, whereas the recipient fetus predominantly shows changes related to volume overload and cardiac dysfunction [6,7]. In sFGR, decreased levels of amino acids, glucose, and lipids have been observed, reflecting placental insufficiency, along with increased markers of oxidative stress [8]. However, the vast majority of these studies have focused on antenatal biofluids. The postnatal metabolic status of newborns from twin pregnancies complicated by these conditions remains largely unexplored.

The placenta is the primary interface for amino acid transfer from mother to fetus. Several transporter systems are involved, including System A (neutral amino acids), System L (branched-chain and aromatic amino acids), and System y^+^ (cationic amino acids) [9,10]. In placental insufficiency, the expression and activity of these transporters are downregulated, leading to reduced fetal amino acid availability. This is particularly pronounced in sFGR, where the smaller twin exhibits lower concentrations of essential amino acids and impaired protein synthesis [11]. In TTTS, the donor twin is similarly affected by insufficient placental share, whereas the recipient twin may experience volume overload and altered amino acid handling [7]. These differences create distinct metabolic profiles between co-twins, which may persist into the postnatal period.

After birth, twins undergo a period of rapid metabolic adaptation. In MC twins, the shared placenta and vascular anastomoses may have long-lasting effects on metabolic regulation, even in the absence of overt TTTS. Studies have shown that MC twins exhibit differences in glucose and lipid metabolism compared with DC twins, potentially reflecting intrauterine programming [12]. However, data on amino acid profiles in the postnatal period remain scarce. Our study addresses this gap by comparing intra-pair discordance in amino acid levels between MC and DC twins at birth and at the corrected full-term age.

Oxidative stress is a key contributor to the pathogenesis of both TTTS and sFGR. In MC pregnancies, unbalanced blood flow through vascular anastomoses can cause episodes of hypoxia–reoxygenation, leading to the generation of reactive oxygen species [13]. The resulting oxidative damage affects placental function and fetal metabolism, including amino acid homeostasis. However, direct measurements of oxidative stress markers in amniotic fluid and cord blood remain limited. In our study, we hypothesised that oxidative stress may also leave a detectable footprint in the postnatal amino acid profile, which could be captured by the intra-pair difference approach.

Dried blood spots (DBSs) represent an ideal biomaterial for neonatal screening and retrospective investigations, as their collection is minimally invasive, samples are stable during storage, and they allow the analysis of a wide range of metabolites [14]. A key methodological challenge when comparing dichorionic (DC) and MC twins lies in their genetic and placental heterogeneity. DC twins are dizygotic and have two separate placentas, whereas MC twins are monozygotic and share a single placenta with possible vascular anastomoses [15]. Direct comparison of absolute metabolite concentrations between newborns from different chorionicity types may be confounded by multiple factors (birth weight, gestational age, sex, genetic differences). To address this methodological issue, we applied a calculation of the normalised intra-pair difference (ΔS_twins), which allows elimination of pair-shared factors and isolation of effects attributable to placental type and complications [16].

The aim of this study was to perform a comparative analysis of amino acid profiles in DBSs from newborns of DC and MC twin pregnancies with uncomplicated courses, as well as those complicated by sFGR and TTTS, assessing the difference between co-twins at birth and its dynamics up to the corrected full-term age.

## 2. Results

### 2.1. Clinical Characteristics of the Study Groups

The study included 16 pairs in the control group, 10 pairs with TTTS, and 7 pairs with sFGR, corresponding to 32, 20, and 14 newborns, respectively (Table 1). The groups differed significantly in gestational age, birth weight, head circumference, and body length at birth (all *p* < 0.001), with the control group showing higher values compared to both pathological groups. Apgar scores at 1 and 5 min were higher in the control group than in the TTTS and sFGR groups (*p* = 0.010 for both scores, except for the 1 min Apgar in the control vs. sFGR comparison, where *p* > 0.05 after correction). Sex distribution differed between groups: females predominated in the sFGR group (85.7%), whereas males predominated in the TTTS group (70.0%), *p* = 0.006. As expected, all TTTS cases were monochorionic, whereas only 18.8% of the control group were monochorionic (*p* < 0.001). Maternal anthropometric parameters (body weight, height, weight gain, and body mass index (BMI)) were comparable across groups. Maternal age was significantly higher in the control group compared to the sFGR group (post hoc *p* = 0.044), but did not differ between the control and TTTS groups (*p* = 0.106) or between TTTS and sFGR (*p* = 0.822). The rates of preeclampsia and gestational diabetes mellitus did not differ between groups (*p* = 0.841 and *p* = 0.250, respectively).

### 2.2. Analysis of Amino Acid Profiles

The levels of 23 amino acids were determined in the DBS samples. For each twin pair, the normalised intra-pair difference (ΔS_twins) was calculated, which allowed us to minimise the influence of absolute concentrations dependent on body weight and gestational age. All differences presented below were statistically significant at *p* < 0.05 after Benjamini–Hochberg FDR correction (q < 0.20). Data are presented as median [Q1; Q3].

#### 2.2.1. Effect of Chorionicity Type (DC vs. MC)

At birth (T0), no significant differences in ΔS_twins were observed between DC and MC twins overall, indicating that chorionicity type does not influence metabolic discordance in the absence of complications.

At the corrected full-term age (T1), a trend towards greater discordance for proline was observed in MC pairs compared with DC pairs: median ΔS was 10.38% [6.78; 14.52] vs. 4.9% [2.18; 9.4], respectively (*p* = 0.037) (Table 2).

Analysis of individual dynamics (ΔS_ind) revealed that the change in valine levels from T0 to T1 differed significantly between MC and DC twins: 13.37% [6.34; 27.23] vs. 31.39% [12.98; 40], respectively (*p* = 0.037) (Table 3). Similarly, the change in intra-pair discordance (ΔΔS_twins) for alanine differed between MC and DC pairs: 7.31% [2.84; 10.62] vs. 19.77% [13.89; 26] (*p* < 0.001) (Table 4).

#### 2.2.2. Fetal Pathologies vs. Controls

##### sFGR vs. Control

At birth (T0), the sFGR group exhibited increased intra-pair discordance compared with controls for glycine (20.66% [16.25; 23.95] vs. 5.65% [3.34; 9.36], *p* = 0.007) and alanine (24.76% [24.05; 25.31] vs. 5.5% [2.86; 12.73], *p* = 0.002) (Table 2).

At the corrected full-term age (T1), the sFGR group showed decreased discordance compared with controls for β-alanine (3.22% [1.91; 4.99] vs. 18.01% [10.23; 28.9], *p* = 0.011) and leucine (2.61% [1.35; 3.21] vs. 8.77% [6.02; 15.56], *p* = 0.047) (Table 2).

Analysis of dynamics revealed significant differences between sFGR and controls for alanine (ΔΔS_twins: 33.78% [28.02; 39.5] vs. 8.68% [3.75; 13.7], *p* = 0.003), asparagine (19.95% [17.4; 23.22] vs. 11.17% [5.84; 14.4], *p* = 0.047), citrulline (7.86% [5.43; 8.09] vs. 24.3% [10.48; 34.75], *p* = 0.035), and tyrosine (31.31% [31.1; 36.64] vs. 14.01% [4.9; 24.74], *p* = 0.017) (Table 4).

##### TTTS vs. Control

At birth (T0), the TTTS group showed increased discordance compared with controls for glycine (10.13% [7.01; 15.37] vs. 5.65% [3.34; 9.36], *p* = 0.025), although this value was lower than in the sFGR group (Table 2).

At the corrected full-term age (T1), the TTTS group exhibited increased discordance compared with controls for glutamine (24.91% [16.89; 40] vs. 9.82% [4.73; 16.07], *p* = 0.031) (Table 2).

Analysis of individual dynamics (ΔS_ind) revealed that the change in alanine (18.09% [9.55; 26.41] vs. 8.31% [4.87; 15.51], *p* = 0.036), valine (32.73% [24.46; 40] vs. 15.87% [5.57; 32.89], *p* = 0.008), and glutamic acid (9.54% [7.62; 16.73] vs. 6.37% [3.15; 11.26], *p* = 0.042) was higher in TTTS than in controls, whereas the change in GABA was lower in TTTS than in controls (15.62% [8.92; 25.67] vs. 31.26% [18.25; 50.76], *p* = 0.016) (Table 3).

Analysis of ΔΔS_twins revealed significant differences between TTTS and controls for alanine (14.72% [13.49; 26.06] vs. 8.68% [3.75; 13.7], *p* = 0.004), α-aminobutyric acid (9.21% [2.13; 10.83] vs. 21.97% [11.44; 30.86], *p* = 0.041), and aspartic acid (7.29% [3.84; 10.41] vs. 24.53% [14.3; 38.86], *p* = 0.003) (Table 4).

#### 2.2.3. Direct Comparison Between TTTS and sFGR

At birth (T0), citrulline significantly distinguished TTTS from sFGR: median ΔS for TTTS was 16.64% [5; 26.64], while for sFGR it was 41.47% [37.04; 41.8] (*p* = 0.018) (Table 2).

At the corrected full-term age (T1), β-alanine discordance differed significantly between TTTS and sFGR: 22.17% [18; 32.27] vs. 3.22% [1.91; 4.99], respectively (*p* = 0.018) (Table 2).

Analysis of individual dynamics (ΔS_ind) revealed significant differences between TTTS and sFGR for valine (32.73% [24.46; 40] vs. 11.53% [6.68; 19.74], *p* = 0.047), leucine (14.4% [7.75; 21.68] vs. 28.3% [22.08; 33.62], *p* = 0.027), and ornithine (24.18% [11; 40.69] vs. 45.82% [42.01; 61.42], *p* = 0.015) (Table 3).

#### 2.2.4. Multivariate Analysis

PCA and PLS-DA score plots showed substantial overlap among the Control, TTTS, and sFGR groups at both T0 and T1 (Supplementary Figures S1–S4). No clear group separation was observed. The first two principal components explained 30.0% and 32.3% of the variance at T0 and T1, respectively. PLS-DA also did not reveal distinct clusters, although a trend towards separation was noted. VIP analysis identified valine, glycine, β-alanine, ornithine, asparagine, α-aminobutyric acid, methionine, γ-aminobutyric acid, glutamine, alanine, tyrosine, arginine, tryptophan at T0 and 3-aminoisobutyric acid, α-aminobutyric acid, asparagine, arginine, ornithine, β-alanine, glutamine, glycine, citrulline, tyrosine at T1 as the main contributors to group discrimination (Supplementary Figures S5 and S6, Supplementary Tables S1 and S2). These findings should be interpreted as exploratory due to the small sample size.

#### 2.2.5. Secondary Analysis: Maternal Complications (PE, GDM)

In the GDM subgroup, increased intra-pair discordance compared with controls was observed at birth for proline (20.18% [11.1; 29.63] vs. 4.58% [3.16; 12.35], *p* = 0.011) and aspartic acid (33.12% [26.3; 44.24] vs. 11.27% [7.18; 27.84], *p* = 0.043), and at the corrected full-term age for serine (27.79% [26.38; 28.41] vs. 11.79% [3.06; 18.25], *p* = 0.047) and threonine (2.91% [0.82; 4.78] vs. 13.84% [8.77; 20.04], *p* = 0.022) (Table 2).

In the PE subgroup, differences from controls were found at birth for 3-aminoisobutyric acid (11.04% [5.06; 11.86] vs. 24.92% [11.87; 41.23], *p* = 0.024) (Table 2).

Direct comparison between PE and GDM at the corrected full-term age revealed differences for threonine (26.34% [15.57; 54.71] vs. 2.91% [0.82; 4.78], *p* = 0.017), ornithine (44.76% [39.54; 51.59] vs. 9.64% [5.23; 15.43], *p* = 0.030), and glutamic acid (5.76% [5.71; 9] vs. 2.55% [2.21; 2.98], *p* = 0.004) (Table 2).

Analysis of dynamics revealed significant differences between PE and GDM for ornithine (ΔΔS_twins: 40.22% [39.24; 47.12] vs. 7% [2.94; 13.69], *p* = 0.009) (Table 4). In the GDM subgroup, dynamics differed from controls for proline (ΔS_ind: 29.15% [14.81; 36.13] vs. 12.14% [8.44; 22.03], *p* = 0.038) and tyrosine (51.9% [33.51; 63.2] vs. 17.34% [9.51; 29.07], *p* < 0.001) (Table 3), as well as for alanine, proline, and aspartic acid (Table 4). In the PE subgroup, dynamics differed from controls for glycine (ΔS_ind: 37.97% [28.53; 53.87] vs. 25.44% [17.12; 38.16], *p* = 0.049) and glutamic acid (12.52% [8.78; 17.25] vs. 7.56% [2.86; 9.98], *p* = 0.021) (Table 3), as well as for glycine in ΔΔS_twins (Table 4).

## 3. Discussion

To the best of our knowledge, this pilot study is the first to apply the intra-pair difference index (ΔS_twins) for comparative metabolomic profiling of newborns from dichorionic and monochorionic twin pregnancies. This approach allows us to mitigate the influence of genetic heterogeneity (twins are dizygotic in DC pairs and monozygotic in MC pairs), as well as variability in absolute concentrations arising from differences in birth weight and gestational age [16]. Direct comparisons of absolute metabolite levels between monozygotic and dizygotic twins have rarely been performed in the literature, underscoring the methodological value of our strategy.

Our data demonstrate that, in the absence of obstetric complications, intra-pair amino acid discordance is independent of chorionicity type. This suggests that a shared placenta with vascular anastomoses does not create substantial metabolic differences between twins under favourable pregnancy conditions. However, at the second time point (corrected full-term age), MC pairs exhibited greater discordance for proline, and the normalisation dynamics for valine were delayed. Proline plays a key role in collagen synthesis, tissue repair, and antioxidant defence [17]. We hypothesise that even in the absence of clinical TTTS, MC placentation is associated with a degree of chronic hypoxia or uneven blood flow, which leaves a “metabolic footprint” in the postnatal period, reflected in altered proline and valine homeostasis. Valine, as a branched-chain amino acid, participates in energy metabolism and neurotransmitter synthesis; its delayed normalisation may indicate a longer adaptive period for muscle and neural metabolism in MC newborns.

In sFGR, the increased discordance for glycine and alanine at birth is consistent with published data on placental insufficiency, which is accompanied by reduced transport of neutral amino acids [8]. Glycine is not only a structural protein component but also an important neurotransmitter and a participant in haem synthesis [18]. Glycine deficiency may arise from insufficient placental transport and increased utilisation for haemoglobin synthesis under hypoxic conditions.

Interestingly, by the corrected full-term age, β-alanine discordance in the sFGR group was significantly lower than in controls (3.22% vs. 18.01%, *p* = 0.011). β-Alanine is a precursor of carnosine, an intracellular buffer and antioxidant [19]. The reduction in inter-twin differences for this metabolite may indicate a possible depletion of antioxidant reserves in the smaller twin against the background of prolonged placental insufficiency, rather than their compensatory upregulation. This aligns with the notion that oxidative stress persists in sFGR, but protective mechanisms become exhausted by the time of full-term correction. However, direct measurements of carnosine and other antioxidants are needed to confirm this hypothesis. This interpretation is preliminary and requires experimental verification.

One of the key distinguishing features between TTTS and sFGR was citrulline discordance, which was significantly higher in sFGR than in TTTS. Citrulline is a product of the reaction catalysed by NO synthases, including the endothelial isoform (eNOS), which is highly active in the vascular endothelium of newborns [20,21]. Increased citrulline discordance in sFGR may reflect asymmetry of endothelial function associated with placental insufficiency. Conversely, in TTTS, despite volume overload in the recipient and hypovolaemia in the donor, citrulline discordance was less pronounced, suggesting alternative regulatory mechanisms of the NO synthase pathway in this syndrome.

Analysis of individual dynamics (change in ΔS_twins from birth to corrected full-term age, ΔS_ind) revealed the most prominent differences in TTTS (alanine, GABA, valine, glutamic acid) and in sFGR (valine, leucine, ornithine). The dynamic parameter likely better reflects the long-term consequences of intrauterine pathology than a static measure at birth. For instance, GABA dynamics in TTTS were significantly lower than in controls (15.62% vs. 31.26%, *p* = 0.016). GABA is the primary inhibitory neurotransmitter in adults; however, in the early postnatal period, its effect may be excitatory due to high intracellular Cl^−^ levels [22,23]. The maturation of the GABAergic system critically depends on the functional activity of GABA transporters, which regulate extracellular GABA levels and shape the developmental switch in GABA signalling [24]. The reduced GABA dynamics in TTTS newborns may indicate impaired maturation of the GABAergic system, potentially associated with risks of neurological impairment. This observation is consistent with the known risks of adverse neurological outcomes in children with TTTS [25] and warrants further investigation.

The results concerning GDM and PE also deserve attention, although these subgroups were small. The increased intra-pair difference for proline and aspartic acid in GDM may reflect the influence of hyperinsulinaemia on neutral amino acid transport and gluconeogenesis. It is well established that fetuses of diabetic mothers exhibit altered amino acid ratios, contributing to macrosomia or, conversely, growth restriction depending on glycaemic control [26]. The differences in threonine and ornithine between GDM and PE may serve as differential metabolic markers of these maternal complications in the offspring.

Several limitations of this work must be acknowledged. The analysis was limited to amino acids; other metabolite classes (acylcarnitines, organic acids, lipids) were not investigated. Expanding the metabolomic panel is a priority for future work. The small sample size, particularly in the PE and GDM subgroups, limits the feasibility of multivariate analysis and increases the risk of type II error. We did not stratify TTTS cases by stage, nor did we analyse donors and recipients separately. The absence of long-term follow-up data precludes assessment of the clinical relevance of the observed metabolic shifts for child health. Nevertheless, our methodological approach (ΔS_twins combined with FDR correction) renders the findings more robust than direct comparisons of absolute concentrations. The less stringent FDR threshold was a conscious trade-off given the hypothesis-generating nature of this study; all observed associations must be validated in independent cohorts using stricter statistical thresholds.

## 4. Materials and Methods

### 4.1. Study Design

The study was conducted in accordance with the Declaration of Helsinki and was approved by the local ethics committee (protocol No. 4 2021/04). This study included DBS samples collected from 66 newborns (33 twin pairs), comprising both term and preterm twins born between 2018 and 2022. Inclusion criteria were confirmed chorionicity type (verified by histopathological examination) and availability of complete clinical data. The groups were allocated as follows: Group 1 (control)—16 pairs of DC and MC twins with uncomplicated pregnancies; Group 2 (TTTS)—10 pairs diagnosed according to the Quintero criteria (stages I–IV) [27]; Group 3 (sFGR)—7 pairs with a birth weight discordance > 20% and the smaller twin having a birth weight below the 10th percentile according to population norms. Additional comparisons were performed between subgroups with PE (7 cases) and GDM (7 cases), drawn from the overall cohort. Additional comparisons with PE and GDM subgroups were exploratory and were not the primary aim of the study. Blood samples were collected from the newborn’s heel onto filter paper within the first 24–48 h of life (time point T0, “birth”) and again upon reaching the corrected full-term age (40 weeks) for preterm infants, or after 14 days for term infants (time point T1). The choice of time point T0 is based both on neonatal screening standards and on the need to capture early postnatal metabolic shifts that may reflect intrauterine pathology.

### 4.2. Extraction of Amino Acids from DBSs

A 6 mm diameter disc was punched from each DBS. Sample preparation involved the following steps: the disc was placed into a 1.5 mL microcentrifuge tube, and 480 µL of chloroform:methanol (2:1, *v*/*v*) was added, followed by 150 µL of deionised water. The mixture was vigorously shaken for 10 min and then centrifuged at 15,000× *g* for 10 min at room temperature to achieve phase separation. The lower (chloroform-methanol) phase was collected, and the remaining sample was re-extracted by adding 250 µL of chloroform:methanol (2:1, *v*/*v*), shaking for 10 min, and centrifuging again. A 150 µL aliquot of the upper aqueous-methanol phase was transferred and dried under a nitrogen stream at 60 °C for 30 min. For derivatisation, 200 µL of 0.1 M hydrochloric acid in butanol was added to the dried residue, mixed for 3 min, centrifuged at 13,000× *g* for 15 s, and incubated at 60 °C for 15 min. After another centrifugation, the sample was again dried under nitrogen at 60 °C for 30 min. The dried residue was dissolved in 300 µL of acetonitrile:water (1:1, *v*/*v*), mixed for 5 min, and centrifuged at 13,000× *g* for 15 s. Finally, 200 µL of the supernatant was transferred into a vial for analysis.

### 4.3. HPLC–MS/MS Analysis

Chromatographic separation was performed on an Agilent 1260 II system (Agilent, Waldbronn, Germany) equipped with an Agilent Zorbax Eclipse XDB-C18 column (100 mm × 2.1 mm, 1.8 µm, Agilent, Santa Clara, CA, USA). The mobile phases consisted of (A) 10 mM ammonium acetate in water and (B) 100% acetonitrile. The gradient program was as follows: 0–0.1 min, 5% B; 0.1–0.2 min, 25% B; 0.2–5 min, 30% B; 5–15 min, 55% B; 15–20 min, 70% B; 20–20.5 min, 95% B; held at 95% B until 25 min; returned to 5% B at 25.5 min; and re-equilibrated until 40 min. The flow rate was 150 µL/min, and the injection volume was 3 µL. Mass spectrometric detection was carried out using an Agilent 6460 triple quadrupole mass spectrometer (Agilent, Waldbronn, Germany) operating in positive ion mode with an electrospray ionization (ESI) source. The ionization parameters were a drying gas temperature of 150 °C, drying gas flow of 10 L/min, nebulizer pressure of 2.76 bar, sheath gas temperature of 400 °C, sheath gas flow of 10 L/min, and capillary voltage of 2000 V.

### 4.4. Metabolomic Data Analysis

Statistical analysis was performed using the R-script. For each amino acid, the normalised intra-pair difference was calculated according to the following formula:

(1) $$\Delta S\text{\_}\mathrm{twins}=\frac{\left|S\text{\_}\mathrm{twin}1\;-S\text{\_}\mathrm{twin}2\right|}{(S\_\mathrm{twin}1+S\_\mathrm{twin}2)/2}\times 100\%,$$

where S_twin1 is the amino acid level in the first newborn of the pair (arbitrarily defined as the “larger” twin in the control group, the recipient in TTTS, or the heavier twin in sFGR), and S_twin2 is the level in the second twin (the “smaller” twin, the donor in TTTS, or the lighter twin in sFGR).

Additionally, we assessed the individual dynamics of each metabolite per child (ΔS_ind) and the change in intra-pair discordance over the observation period (ΔΔS_twins):

(2) $$\Delta S\text{\_}\mathrm{ind}=\frac{\left|S\text{\_}\mathrm{twin}\text{\_}T1\;-S\text{\_}\mathrm{twin}\text{\_}T0\right|}{(S\text{\_}\mathrm{twin}\text{\_}T1+S\text{\_}\mathrm{twin}\text{\_}T0)/2}\times 100\%,$$

(3) $$\Delta \Delta S\text{\_}\mathrm{twins}=\frac{\left|\Delta S\text{\_}\mathrm{twins}\text{\_}T1\;-\Delta S\text{\_}\mathrm{twins}\text{\_}T0\right|}{(\Delta S\text{\_}\mathrm{twins}\text{\_}T1+\Delta S\text{\_}\mathrm{twins}\text{\_}T0)/2}\times 100\%$$

In the above formulae:

S_twin_T0 and S_twin_T1 are the amino acid levels for the same twin at time points T0 and T1, respectively;

ΔS_twins_T0 and ΔS_twins_T1 are the intra-pair differences calculated using Formula (1) at the corresponding time points.

Normality of the distribution was tested using the Kolmogorov–Smirnov test; since most variables were non-normally distributed, group comparisons were performed using the non-parametric Mann–Whitney U test. Given the multiple comparisons (23 amino acids, several groups, and time points), the Benjamini–Hochberg false discovery rate (FDR) correction was applied with a threshold of *q* < 0.20. The *q* < 0.20 threshold was chosen given the pilot nature of the study and the need to minimise the risk of missing potentially significant metabolic signals. The results were interpreted as hypothesis-generating.

### 4.5. Multivariate Analysis

To explore global metabolic patterns, principal component analysis (PCA) and partial least squares discriminant analysis (PLS-DA) were performed separately for T0 and T1. Amino acid data were log10-transformed and autoscaled. PCA was used to assess unsupervised clustering. PLS-DA with two components was used to model group separation; variable importance in projection (VIP) scores > 1 were considered potentially discriminating. Given the exploratory nature and small sample size, multivariate results were interpreted cautiously and not used for formal classification.

### 4.6. Clinical Data Analysis

To characterise the study groups, the following maternal parameters were analysed: age, body weight, height, weight gain, BMI, presence of PE, and GDM. Neonatal parameters included gestational age, birth weight, head circumference, body length, Apgar scores at 1 and 5 min, sex, and chorionicity type. Continuous variables are presented as medians with interquartile ranges (Q1–Q3) due to non-normal distribution, as verified by the Shapiro–Wilk test. Comparisons among the three groups were performed using the non-parametric Kruskal–Wallis test; when significant differences were found, post hoc pairwise comparisons were conducted with Bonferroni correction. Categorical variables were compared using the chi-square test (or Fisher’s exact test when expected frequencies were less than 5). Statistical significance was set at *p* < 0.05. All calculations were performed in the Python environment (version 3.13.3) using the scipy (version 1.18.0), and statsmodels (version 0.15.0) libraries.

## 5. Conclusions

The use of the intra-pair difference index (ΔS_twins) allowed us to minimise the influence of genetic and placental heterogeneity and to identify metabolic patterns associated with sFGR and TTTS. In uncomplicated pregnancies, chorionicity type does not affect discordance at birth; however, MC twins exhibit delayed postnatal adaptation (normalisation of valine and proline), which warrants clinical attention. Potential metabolic signatures were preliminarily identified: for sFGR, increased discordance for glycine and alanine at birth and decreased discordance for β-alanine at the corrected full-term age; for TTTS, low citrulline discordance at birth, which significantly distinguishes it from sFGR where this parameter was elevated. Additionally, newborns with TTTS showed significantly lower dynamics of GABA and glutamic acid from birth to the corrected full-term age compared with the control group, which may reflect impaired adaptive processes in neurotransmitter systems. However, given the pilot nature of this study and the limited sample size, both the identified patterns and the proposed approach require independent validation in larger cohorts. Our findings provide a basis for generating hypotheses on the pathogenesis of metabolic disturbances in TTTS and sFGR, but their implementation into routine screening is premature until the prognostic value is confirmed in prospective studies.

## Figures and Tables

**Table 1:** Clinical characteristics of the cohort.

Parameter	Control (n_m_ = 16, n_n_ = 32)	TTTS (n_m_ = 10, n_n_ = 20)	sFGR (n_m_ = 7, n_n_ = 14)	*p*-Value
Maternal parameters				
Age, years	38.0 [33.3; 39.3]	32.0 [29.3; 34.8]	33.0 [27.0; 34.0]	<0.05 ^1^
Body weight, kg	70.7 [66.5; 80.8]	75.0 [66.0; 81.7]	85.0 [71.8; 88.9]	0.192 ^2^
Height, cm	167.5 [162.8; 173.0]	161.0 [155.3; 167.5]	168.0 [162.5; 169.5]	0.215 ^2^
Weight gain, kg	12.0 [10.8; 15.0]	12.0 [11.0; 14.5]	11.0 [9.0; 16.0]	0.914 ^2^
BMI, kg/m^2^	26.1 [24.0; 28.1]	27.7 [24.2; 33.3]	29.4 [27.8; 31.3]	0.475 ^2^
Preeclampsia, *n* (%)	4 (25.0)	2 (20.0)	1 (14.3)	0.841 ^3^
GDM, *n* (%)	3 (18.8)	1 (10.0)	3 (42.9)	0.250 ^3^
Neonatal parameters				
Gestational age, weeks	34.0 [32.9; 36.0]	32.0 [32.0; 33.0]	32.0 [31.7; 33.1]	<0.001
Birth weight, g	2025 [1746; 2388]	1755 [1198; 1893]	1480 [1035; 1810]	<0.001
Head circumference, cm	32.0 [31.8; 33.0]	30.5 [28.0; 31.3]	29.0 [27.3; 31.0]	<0.001
Body length, cm	46.0 [42.8; 48.0]	42.5 [39.3; 44.0]	40.0 [37.3; 41.0]	<0.001
Apgar score, 1 min	7.0 [7.0; 8.0]	7.0 [6.0; 7.0]	7.0 [6.3; 7.0]	0.010
Apgar score, 5 min	8.0 [8.0; 8.3]	8.0 [7.0; 8.0]	7.5 [7.0; 8.0]	0.010
Male sex, *n* (%)	14 (43.8)	14 (70.0)	2 (14.3)	0.006
Monochorionic placentation, *n* (%)	6 (18.8)	20 (100)	12 (85.7)	<0.001

Data are presented as median [Q1–Q3] for continuous variables and *n* (%) for categorical variables. n_m_—number of mothers, n_n_—number of newborns. ^1^ Overall *p* < 0.05; pairwise comparison significant between Control and sFGR groups (*p*-adj = 0.044). ^2^ No significant differences between groups for maternal anthropometric parameters (all *p* > 0.05 in post hoc tests). ^3^ No significant differences for preeclampsia and GDM (chi-square test).

**Table 2:** Intra-pair discordance (ΔS_twins) in amino acid levels at birth (T0) and at corrected full-term age (T1).

Comparison (Group 1 vs. Group 2)	Time Point	Amino Acid	ΔS_twins, %		*p*-Value ^1^
			Group 1	Group 2	
Control vs. GDM	T0	Proline	4.58 [3.16; 12.35]	20.18 [11.1; 29.63]	0.011
Control vs. GDM	T0	Aspartic acid	11.27 [7.18; 27.84]	33.12 [26.3; 44.24]	0.043
Control vs. PE	T0	3-Aminoisobutyric acid	24.92 [11.87; 41.23]	11.04 [5.06; 11.86]	0.024
Control vs. TTTS	T0	Glycine	5.65 [3.34; 9.36]	10.13 [7.01; 15.37]	0.025
Control vs. sFGR	T0	Glycine	5.65 [3.34; 9.36]	20.66 [16.25; 23.95]	0.007
Control vs. sFGR	T0	Alanine	5.5 [2.86; 12.73]	24.76 [24.05; 25.31]	0.002
TTTS vs. sFGR	T0	Citrulline	16.64 [5; 26.64]	41.47 [37.04; 41.8]	0.018
MC vs. DC	T1	Proline	4.9 [2.18; 9.4]	10.38 [6.78; 14.52]	0.037
Control vs. GDM	T1	Serine	11.79 [3.06; 18.25]	27.79 [26.38; 28.41]	0.047
PE vs. GDM	T1	Threonine	26.34 [15.57; 54.71]	2.91 [0.82; 4.78]	0.017
PE vs. GDM	T1	Ornithine	44.76 [39.54; 51.59]	9.64 [5.23; 15.43]	0.030
PE vs. GDM	T1	Glutamic acid	5.76 [5.71; 9]	2.55 [2.21; 2.98]	0.004
Control vs. TTTS	T1	Glutamine	9.82 [4.73; 16.07]	24.91 [16.89; 40]	0.031
Control vs. sFGR	T1	β-Alanine	18.01 [10.23; 28.9]	3.22 [1.91; 4.99]	0.011
Control vs. sFGR	T1	Leucine	8.77 [6.02; 15.56]	2.61 [1.35; 3.21]	0.047
TTTS vs. sFGR	T1	β-Alanine	22.17 [18; 32.27]	3.22 [1.91; 4.99]	0.018
Control vs. GDM	T0	Proline	4.58 [3.16; 12.35]	20.18 [11.1; 29.63]	0.011

^1^ All presented comparisons remained significant after Benjamini–Hochberg FDR correction (q < 0.20); *p*-values are raw.

**Table 3:** Individual dynamics of amino acid levels (ΔS_ind) in each newborn from birth to corrected full-term age.

Comparison (Group 1 vs. Group 2)	Amino Acid	ΔS_ind, %		*p*-Value ^1^
		Group 1	Group 2	
MC vs. DC	Valine	13.37 [6.34; 27.23]	31.39 [12.98; 40]	0.037
Control vs. GDM	Proline	12.14 [8.44; 22.03]	29.15 [14.81; 36.13]	0.038
Control vs. GDM	Tyrosine	17.34 [9.51; 29.07]	51.9 [33.51; 63.2]	<0.001
Control vs. PE	Glycine	25.44 [17.12; 38.16]	37.97 [28.53; 53.87]	0.049
Control vs. PE	Glutamic acid	7.56 [2.86; 9.98]	12.52 [8.78; 17.25]	0.021
Control vs. TTTS	Alanine	8.31 [4.87; 15.51]	18.09 [9.55; 26.41]	0.036
Control vs. TTTS	GABA	31.26 [18.25; 50.76]	15.62 [8.92; 25.67]	0.016
Control vs. TTTS	Valine	15.87 [5.57; 32.89]	32.73 [24.46; 40]	0.008
Control vs. TTTS	Glutamic acid	6.37 [3.15; 11.26]	9.54 [7.62; 16.73]	0.042
TTTS vs. sFGR	Valine	32.73 [24.46; 40]	11.53 [6.68; 19.74]	0.047
TTTS vs. sFGR	Leucine	14.4 [7.75; 21.68]	28.3 [22.08; 33.62]	0.027
TTTS vs. sFGR	Ornithine	24.18 [11; 40.69]	45.82 [42.01; 61.42]	0.015

^1^ All presented comparisons remained significant after Benjamini–Hochberg FDR correction (q < 0.20); *p*-values are raw.

**Table 4:** Change in intra-pair discordance (ΔΔS_twins) from birth to corrected full-term age.

Comparison (Group 1 vs. Group 2)	Amino Acid	ΔΔS_twins, %		*p*-Value ^1^
		Group 1	Group 2	
MC vs. DC	Alanine	7.31 [2.84; 10.62]	19.77 [13.89; 26]	<0.001
Control vs. GDM	Alanine	11.27 [5.28; 16.51]	26.77 [17.02; 32.23]	0.047
Control vs. GDM	Proline	5.59 [2.92; 9.77]	21.53 [16.48; 27.16]	0.002
Control vs. GDM	Aspartic acid	11.11 [3.44; 22.51]	41.33 [28.86; 45.51]	0.022
Control vs. PE	Glycine	8.33 [6.36; 16.58]	24.74 [21.55; 29.67]	0.024
PE vs. GDM	Ornithine	40.22 [39.24; 47.12]	7 [2.94; 13.69]	0.009
Control vs. TTTS	Alanine	8.68 [3.75; 13.7]	14.72 [13.49; 26.06]	0.004
Control vs. TTTS	α-Aminobutyric acid	21.97 [11.44; 30.86]	9.21 [2.13; 10.83]	0.041
Control vs. TTTS	Aspartic acid	24.53 [14.3; 38.86]	7.29 [3.84; 10.41]	0.003
Control vs. sFGR	Alanine	8.68 [3.75; 13.7]	33.78 [28.02; 39.5]	0.003
Control vs. sFGR	Asparagine	11.17 [5.84; 14.4]	19.95 [17.4; 23.22]	0.047
Control vs. sFGR	Citrulline	24.3 [10.48; 34.75]	7.86 [5.43; 8.09]	0.035
Control vs. sFGR	Tyrosine	14.01 [4.9; 24.74]	31.31 [31.1; 36.64]	0.017

^1^ All presented comparisons remained significant after Benjamini–Hochberg FDR correction (q < 0.20); *p*-values are raw.

## Data Availability

The original contributions presented in this study are included in the article. Further inquiries can be directed at the corresponding author.

## Supplementary Materials

The following supporting information can be downloaded at: https://www.mdpi.com/article/10.3390/ijms27219428/s1.

## Abbreviations

The following abbreviations are used in this manuscript:

BMI	Body mass index
DBS	Dried blood spot
DC	Dichorionic
ESI	Electrospray ionization
FDR	False discovery rate
GABA	Gamma-aminobutyric acid
GDM	Gestational diabetes mellitus
HPLC	High-performance liquid chromatography
MC	Monochorionic
MS	Mass spectrometry
PE	Preeclampsia
(s)FGR	(selective) Fetal growth restriction
TTTS	twin-to-twin transfusion syndrome
