# Supplementary material for: Comparative Metabolomic Profiling of Amino Acids in Dried Blood Spots from Dichorionic and Monochorionic Twins Complicated by Selective Fetal Growth Restriction and Twin-to-Twin Transfusion Syndrome
Source: Int J Mol Sci. 2026 Oct 22;27(21):9428. doi: 10.3390/ijms27219428 (PMC13762689; doi:10.3390/ijms27219428)
Supplement: Supplementary file 1 [file ijms-27-09428-s001.zip › ijms-4585640-supplementary.pdf]

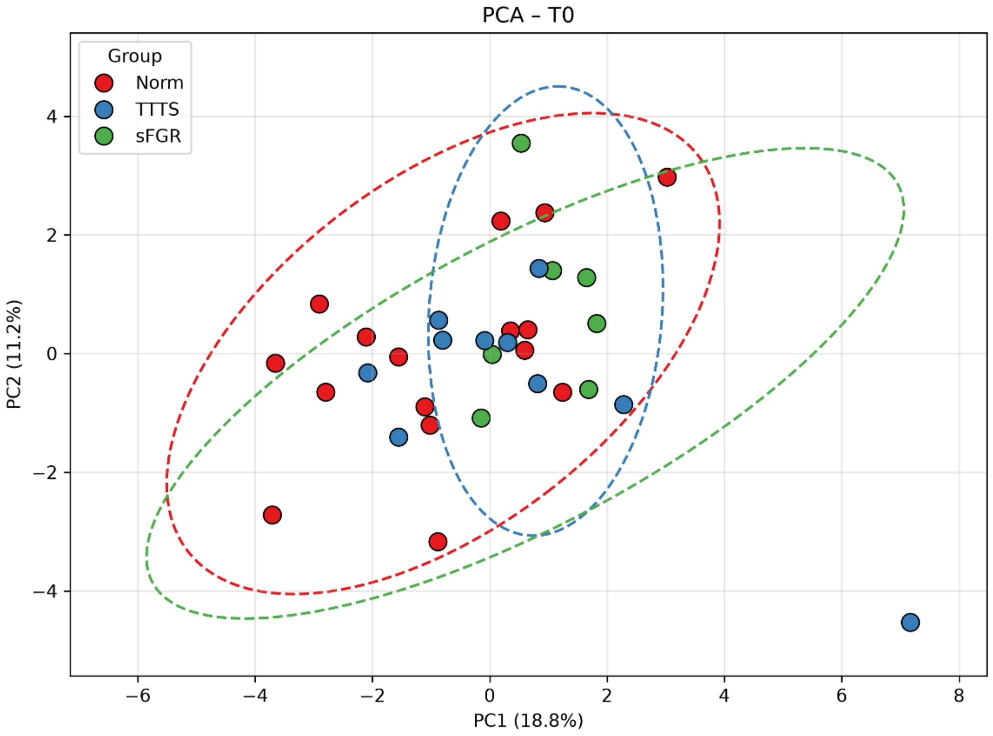

Figure S1. PCA score plot of amino acid profiles in dried blood spots from Control, TTTS, and sFGR twin pairs at birth (T0). Ellipses represent 95% confidence intervals

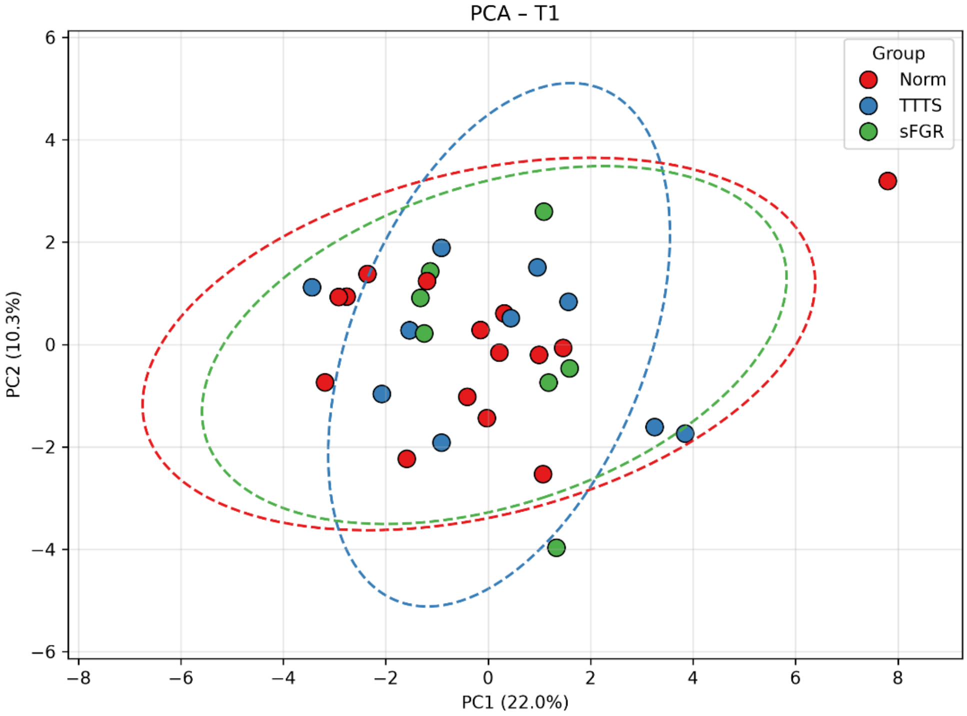

Figure S2. PCA score plot of amino acid profiles in dried blood spots from Control, TTTS, and sFGR twin pairs at birth (T1). Ellipses represent 95% confidence intervals

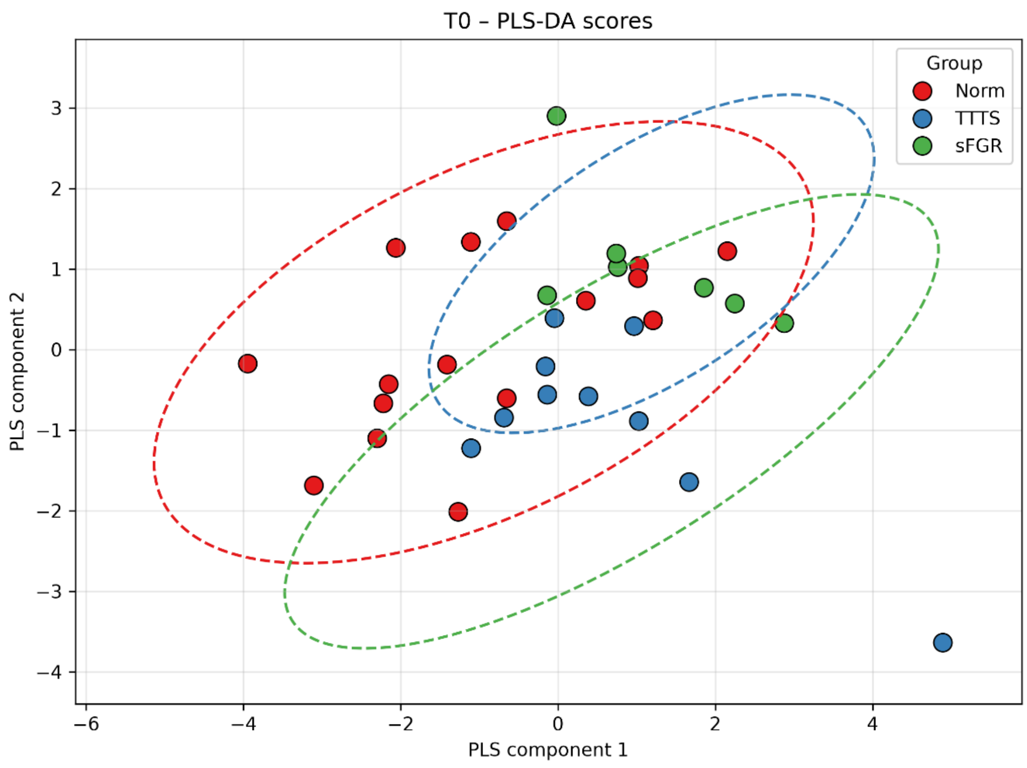

Figure S3. PLS-DA score plot of amino acid profiles in dried blood spots from Control, TTTS, and sFGR twin pairs at birth (T0). Ellipses represent 95% confidence intervals

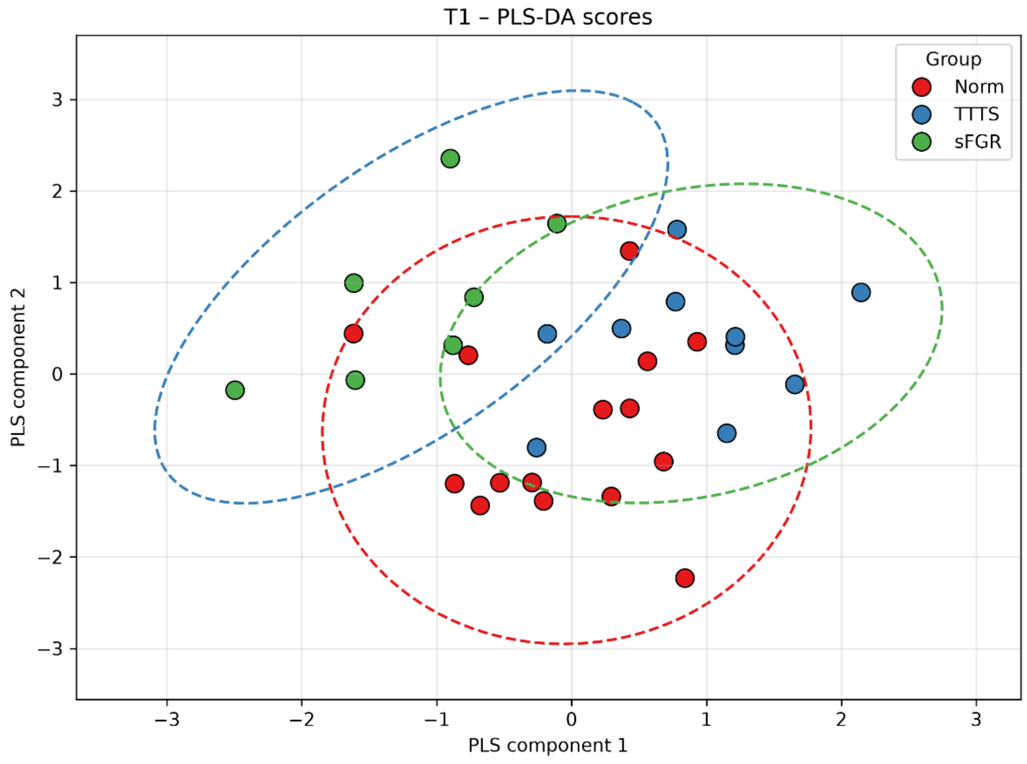

Figure S4. PLS-DA score plot of amino acid profiles in dried blood spots from Control, TTTS, and sFGR twin pairs at birth (T1). Ellipses represent 95% confidence intervals

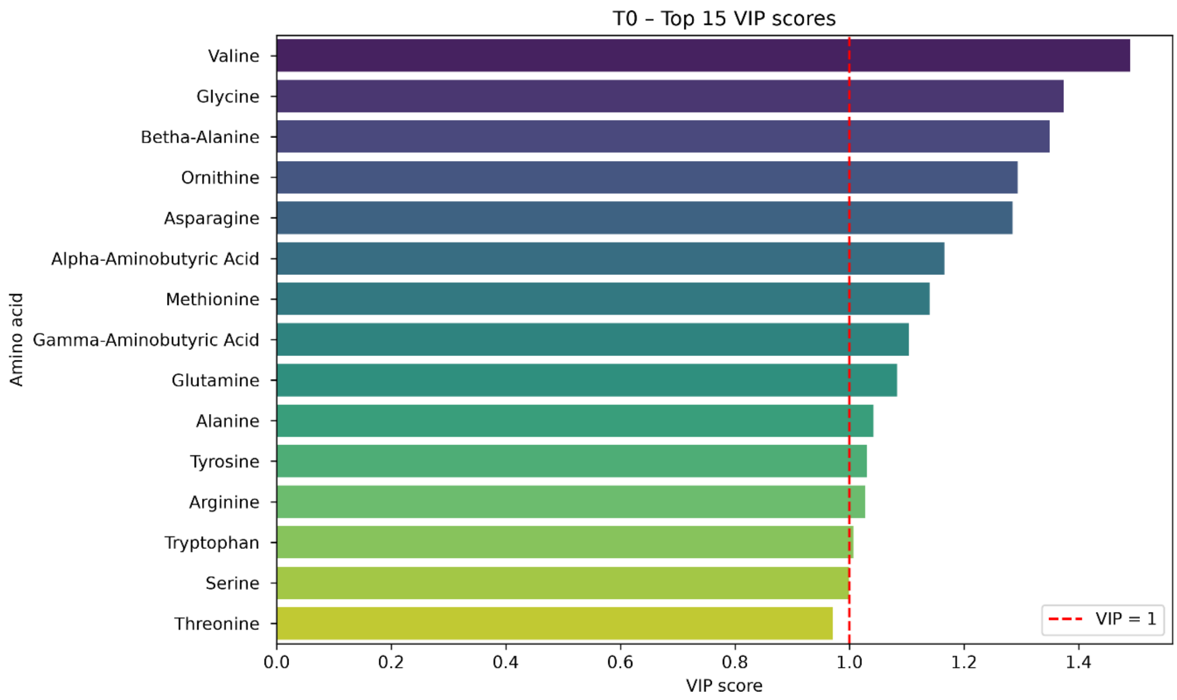

Figure S5. VIP scores for PLS-DA model at T0 (top 15 amino acids; VIP > 1 indicated by red dashed line)

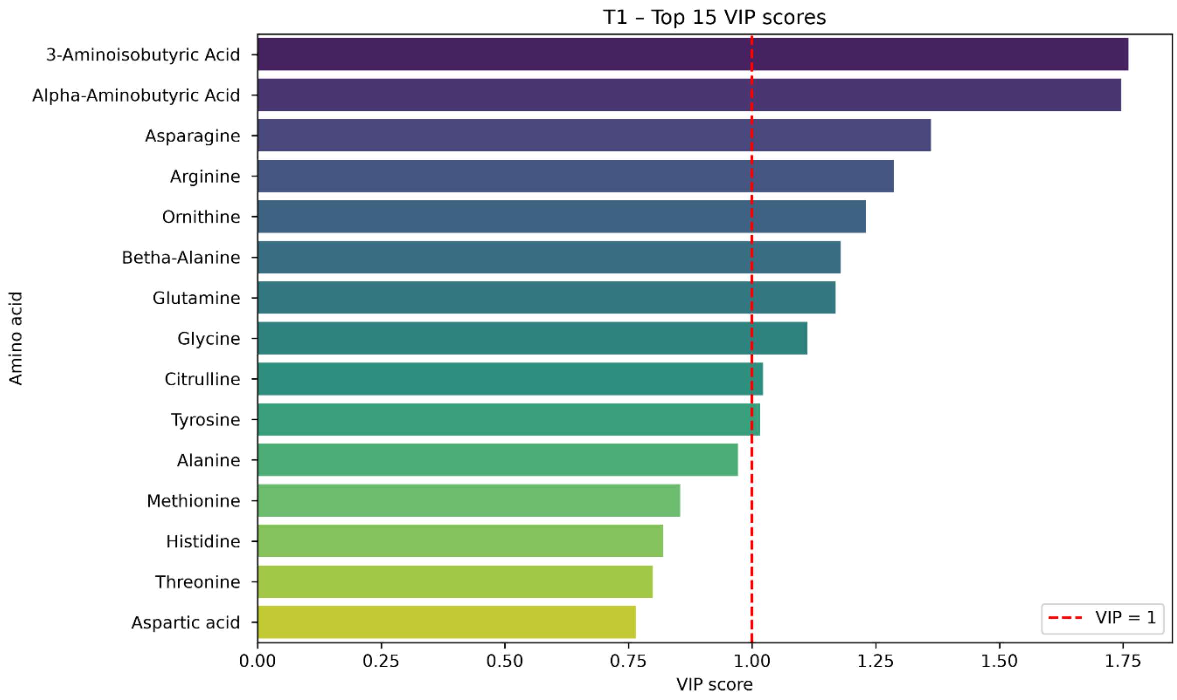

Figure S6. VIP scores for PLS-DA model at T1

Table S1. VIP scores for all amino acids at T0

Amino Acid	VIP
Valine	1.48924
Glycine	1.373931
β-Alanine	1.349348
Ornithine	1.293932
Asparagine	1.284801
α-Aminobutyric Acid	1.165713
Methionine	1.139629
γ-Aminobutyric Acid	1.102997
Glutamine	1.082964
Alanine	1.041916
Tyrosine	1.03038
Arginine	1.02753
Tryptophan	1.00696
Serine	0.999155
Threonine	0.971393
Aspartic acid	0.917905
Histidine	0.758765
Leucine	0.692553
Proline	0.582649
Phenylalanine	0.34534
Citrulline	0.27665
3-Aminoisobutyric Acid	0.27607
Glutamic Acid	0.182606

Table S2. VIP scores for all amino acids at T1

Amino Acid	VIP
3-Aminoisobutyric Acid	1.761436
α-Aminobutyric Acid	1.745638
Asparagine	1.3617
Arginine	1.286111
Ornithine	1.230307
β-Alanine	1.179298
Glutamine	1.169012
Glycine	1.111363
Citrulline	1.022194
Tyrosine	1.016104
Alanine	0.970876
Methionine	0.854564
Histidine	0.820427
Threonine	0.799778
Aspartic acid	0.764916
Glutamic Acid	0.755344
Tryptophan	0.692311
Proline	0.661928
Phenylalanine	0.50957
Serine	0.461165
Leucine	0.329519
Valine	0.310745
γ-Aminobutyric Acid	0.148778
